# Supplementary material for: Pathophysiological Significance of Neutrophilic Transfer RNA-Derived Small RNAs in Asymptomatic Moyamoya Disease
Source: Cells. 2021 May 1;10(5):1086. doi: 10.3390/cells10051086 (PMC8147334; doi:10.3390/cells10051086)

| 2D Structure | Local AU | Position |
| --- | --- | --- |

```
210                              235
                          8mer-1a
5'-agATGTCAT-TCCGTAAAA--ACGGAAAa-3' utr
    |:||  ||   ||::  |  | ||||||| 
3'-uuUGCACUAUUGGUGAUGUGAUGCCUUUg-5' tRF
               16151413    7 6 5 4 3 2
             3' Pairing       Seed
```

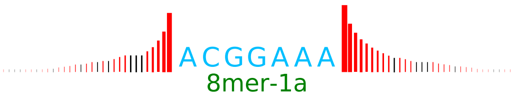


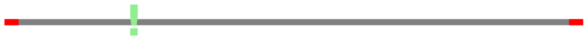

Supplement: Supplementary file 1 [file cells-10-01086-s001.zip › cells-1149161 3rd supplementary materials/Data Supplements/Data Supplement V.zip!/Data supplement 6-mRNAs targets of tsRNAs/tsRNA-Val-AAC-017/Diagrams/tRF-Val-AAC-017_vs_MICT00000164176(CATG00000036588.1).pdf]
